# Supplementary figures and images for: Characterization of a spontaneous microphthalmia-like mutant mouse with isolated ocular defects
Source: PLoS One. 2026 Mar 2;21(3):e0340185. doi: 10.1371/journal.pone.0340185 (PMC12952626; doi:10.1371/journal.pone.0340185)

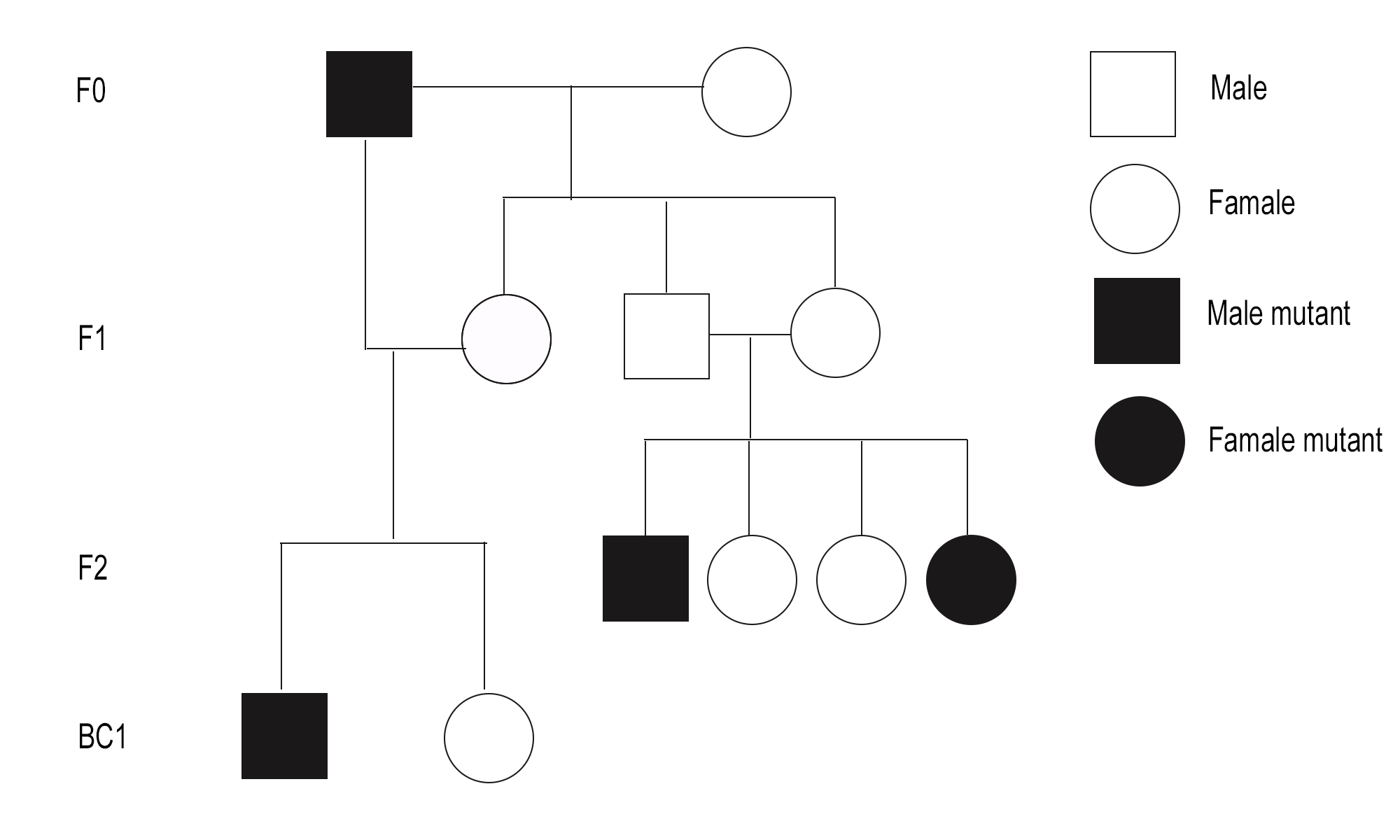

Supplement: S2 Fig — (TIF) [file pone.0340185.s002.tif]
