# Supplementary material for: Characterization of a spontaneous microphthalmia-like mutant mouse with isolated ocular defects
Source: PLoS One. 2026 Mar 2;21(3):e0340185. doi: 10.1371/journal.pone.0340185 (PMC12952626; doi:10.1371/journal.pone.0340185)
Supplement: Attachment — Submitted filename: Response to Reviewers.docx [file pone.0340185.s004.docx]

Dear Editors and Review Experts:

We appreciate your efforts in coordinating the review process and the valuable time and energy invested by the reviewers. We have thoroughly examined and thoughtfully considered the feedback provided by all experts. Their insights were highly constructive and have greatly enhanced the quality of our manuscript. We have revised the manuscript in accordance with the comments and respond to each point as follows. We hope that the revised manuscript meets your expectations.

We have thoroughly revised the original manuscript in accordance with the comments and suggestions provided by the reviewers. The modifications have been highlighted in red underline within the manuscript for your review. We believe that these revisions have significantly improved the logic, rigor, and readability of the paper.

Academic Editor：

1. Please ensure that your manuscript meets PLOS ONE's style requirements, including those for file naming. The PLOS ONE style templates can be found at https://journals.plos.org/plosone/s/file?id=wjVg/PLOSOne_formatting_sample_main_body.pdf and https://journals.plos.org/plosone/s/file?id=ba62/PLOSOne_formatting_sample_title_authors_affiliations.pdf

Author’s Response: We sincerely appreciate your reminder and have meticulously revised the manuscript and supplementary materials to fully comply with PLOS ONE’s style requirements, including file naming conventions, formatting of headings, citations, and other key elements. All manuscript-related files have been renamed in strict adherence to PLOS ONE guidelines and corrected all citations. Placed each caption directly after the paragraph of first citation; used bold for figure titles.

2. Thank you for stating the following financial disclosure:
This research was funded by “The general funding program projects of the Joint Fund for 2024(2023-MSLH-079)” of the Department of Science and Technology of Liaoning Province and “Re-search Platform Construction Project (LT232413610013)” of the Liaoning Education Department.
Please state what role the funders took in the study. If the funders had no role, please state: "The funders had no role in study design, data collection and analysis, decision to publish, or preparation of the manuscript."
If this statement is not correct you must amend it as needed.
Please include this amended Role of Funder statement in your cover letter; we will change the online submission form on your behalf.

Author’s Response: Thank you to the editor for this valuable suggestion. We state the role of funders took in the study and have included an update statement in the cover letter. Here, we sincerely apologize for our oversight. There was a typographical error in the grant number we provided: the "T" in "LT232413610013" should be "J". The correct funding acknowledgments are as follows: This research was funded by “The general funding program projects of the Joint Fund for 2024(2023-MSLH-079)” of the Department of Science and Technology of Liaoning Province and “Re-search Platform Construction Project (LJ232413610013)” of the Liaoning Education Department. The funders had no role in the study design, data collection and analysis, decision to publish, or preparation of the manuscript.

3. Please include captions for your Supporting Information files at the end of your manuscript, and update any in-text citations to match accordingly. Please see our Supporting Information guidelines for more information: http://journals.plos.org/plosone/s/supporting-information.

Author’s Response: Thank you for your valuable reminder. We have strictly adhered to the PLOS Supporting Information guidelines and made the required revisions. Specifically, we have added standardized captions for all supporting information files at the end of the manuscript, and updated all in-text citations to ensure consistency with the file names and captions. The revised supporting information captions (included at the end of the manuscript) are as follows:

Supporting Information

S1 Checklist 1 Author Checklist-Full

S2 Fig 1 Schematic diagram of the breeding process of mutant mice. (Lines602-604)

Reviewer #1:

The authors have discovered and bred a spontaneous microphthalmia-like mutant mouse from a set of C57BL/6J by natural screening, but the genotype responsible for the microphthalmia is still unknown.

Author’s Response: We sincerely appreciate your valuable and insightful comment, which points out a critical direction for improving our research. As you noted, the spontaneous microphthalmia-like mutant mouse was screened and bred from the C57BL/6J background, and the causal genotype remains to be identified. We fully agree that clarifying the underlying genetic mechanism is essential to deepen the biological significance of this mutant model. Regrettably, whole-genome sequencing (WGS) has not been performed in the current study, primarily due to two practical considerations: first, our initial work focused on systematically characterizing the phenotypic traits of the mutant (e.g., ocular morphology, retinal development, and functional assessments), which laid a foundation for subsequent genetic studies; second, budget constraints and the need for collaborative resources (e.g., high-throughput sequencing platforms and bioinformatics analysis pipelines) delayed the initiation of genetic mapping.

To address this limitation, we have already taken concrete steps to advance the genetic characterization. We have preserved genomic DNA from homozygous mutant mice and wild-type littermates (n = 6 per group) to ensure sufficient biological replicates for genetic analysis. We are submitting a supplementary grant application to support WGS. Upon securing funding, we will first perform WGS to identify potential variants within genes known to be involved in ocular development. Again, we thank you for highlighting this important gap and guiding the direction of our follow-up work. Your comment has greatly helped us refine our research plan and enhance the rigor and impact of the study.

They present evidence showing that the basic ocular biometric parameters of the eyes of the mutants are smaller than those of wild type controls. Equatorial diameter and lens diameter measured from enucleated eyes show a significant difference. The eyes in live mice were scanned with OCT to measure the anterior chamber depth, but the OCT images could also be used to measure axial length of the eye, and the corneal thickness, which may or not be thinner in the mutants.

Author’s Response: We sincerely appreciate the reviewer’s insightful and valuable suggestion, which we fully endorse as it helps enrich the characterization of the mutant ocular phenotype. As noted, our initial work focused on core ocular biometric parameters (equatorial diameter and lens diameter of enucleated eyes) and anterior chamber depth (measured via OCT in live mice). Regarding the potential measurement of axial length and corneal thickness from OCT images, we encountered a practical limitation: the optical coherence tomography (OCT) system at our experimental animal center is primarily configured for high-resolution imaging of local retinal structures. It lacks the specialized spectral-domain OCT module designed for small animals—characterized by sufficient axial resolution and scanning depth—required to accurately quantify axial length (a parameter demanding full ocular penetration). Consequently, we regret that axial length measurement was not feasible with our current equipment.

However, we fully adopted your suggestion to measure corneal thickness using the existing OCT images. In the revised manuscript, we have supplemented this analysis as follows: Corneal thickness was quantified from OCT images, and statistical comparisons between mutant and wild-type controls were performed. The results revealed no significant difference in corneal thickness between the mutant group and the control group (Fig 5B), indicating that the mutant eyes do not exhibit thinner corneas relative to wild-type littermates. We have made the following supplements in the revised manuscript：Through measurement and statistical analysis of corneal thickness, the results showed that the mutant group did not exhibit significantly thinner corneas compared to the control group (Fig5 B). (Lines335-338).

The authors present evidence that there are no anatomical abnormalities in critical organs outside the eyes, but any description of the overall health of the mutants is lacking in the manuscript.

Author’s Response: We sincerely appreciate the reviewer’s astute observation, which has helped us improve the comprehensiveness of our study by supplementing critical information on the mutants’ systemic health. To address the gap of lacking descriptions on overall health status, we added a description of the overall health status of the mutant mice in the fifth section of the results section：

To verify whether the mutant mice exhibit systemic health differences compared to wild-type controls, we conducted a comprehensive baseline health assessment. We found that that mutant mice and wild-type mice exhibit a well-groomed, sleek coat without lesions and normal exploratory behavior. Vital parameters, such as heart rate and body temperature, remain within established normal ranges. The animal exhibits a healthy appetite and consistent water consumption and stable body weight. Analysis of this baseline assessment demonstrated no statistically significant variation between the groups. (Lines353-360).

Visual function is assessed by the behavioral oculomotor test, which provides an insight into the overall function of the visual pathways, but does not provide any information on specific functions of the retinae of the mutants. An electroretinography (ERG) analysis would provide information on the overall function of the retina, the night vision and cone function of the retinas of the mutants. It is likely that there may be defects in both.

Author’s Response: We sincerely appreciate the reviewer’s insightful and constructive suggestion, which has significantly enhanced the depth of our retinal function characterization. We fully agree that electroretinography (ERG) is a critical tool to dissect specific retinal functions—including overall retinal integrity, night vision (rod-mediated function), and cone-mediated visual function—that cannot be captured by behavioral oculomotor tests alone. Following your guidance, we have supplemented comprehensive ERG analyses in the revised manuscript to address these key points: To further assess the specific visual function of the mutant retina, we performed ERG analysis under dark adaptation conditions using flash intensities of 1.5 cd·s/m² and 3.0 cd·s/m². The mutant mice exhibited abnormal ERG waveforms (Fig 6 A). The a-wave and b-wave amplitudes in the mutant groups were significantly reduced compared to the control group at flash intensities of 1.5 cd·s/m² and 3.0 cd·s/m², respectively (Fig 6 B). The results indicated that the mutant group exhibited significantly compromised visual function compared to the control group. However, the underlying mechanism for the observed visual impairment in mutant mice remains incompletely understood. This functional deficit may originate from intrinsic retinal pathology, or alternatively, result from the physical obstruction of light entry caused by pupillary atresia in mutant mice (Fig 6 C). (Lines339-349) Notably, as mentioned in the revised manuscript, we acknowledge a potential confounding factor: the presence of pupillary atresia in mutant mice (Fig 6C) may physically obstruct light entry into the retina, which could contribute to the observed ERG deficits alongside intrinsic retinal pathology.

In the Discussion section, we have expanded on this point to contextualize the findings: Also, to further characterize retinal function, we performed electroretinography (ERG), which revealed significant visual function impairment in the mutant mice. However, due to the presence of pupil atresia in some mutant mice, the specific cause of visual function impairment remains unclear. (Lines397-401) ERG deficits are consistent with the structural abnormalities identified via HE staining, but may also be partially attributed to pupillary atresia limiting light penetration. Future studies (e.g., after resolving the pupillary obstruction or via genetic validation of the causal gene) will help disentangle the relative contributions of these two factors to retinal functional impairment.

In Fig 5A there are micrographs of HE stained retinae of a mutant eye and control eye. By looking at these it appears that there are some differences in the thickness of some of the retinal layers, the photoreceptor and ONL seem thinner in the mutant, whilst the RPE appears thicker in the mutant. But this can only be solved by actually measuring the layers and make statistical comparisons. OCT images from the retinae of mutants and control could also be used for that purpose, i.e. to measure the thickness of layers, and if they are even across the retina or not. It appears from the micrographs that there is some variability there in the mutant.

Author’s Response: Thank you very much for your suggestions. We basically agree with your point of view. The retinal HE staining micrographs show that the thickness of certain layers of the retina in the mutant group is different from that in the control group. However, in some mutant mice, HE staining revealed severe disorganization of the outer nuclear layer accompanied by densely packed rosettes, precluding accurate thickness measurement; representative images are shown in Fig 7 A. Therefore, we quantified retinal rosettes in mutant mice to assess the severity of retinal pathology. We have made the following supplements in the revised manuscript：Furthermore, the high density of retinal rosettes in certain regions of the mutant mouse retina makes it challenging to accurately measure the thickness of the outer nuclear layer (ONL). Therefore, we quantified the number of retinal rosettes at multiple time points. A pronounced retinal fold is observed near the optic disc, whereas the peripheral retina appears normal. The results indicated that the outer nuclear layer of the mutant group exhibited varying degrees of severe pathological alterations compared to the control group at various time points (Fig 7 C). Based on these results, we speculate that the pathological alterations in this mouse model were confined to the eyes and did not impact other regions

(Lines368-379). In addition, we have added retinal images of HE staining at other time points in Fig 7 A.

We appreciate you highlighting this issue. The figure has been readjusted and supplemented with other statistical results. Furthermore, we conducted additional measurements of RPE thickness and statistical analyses. We have made the following supplements in result: Histological analysis by HE staining demonstrated a significant thickening of the retinal pigment epithelium (RPE) in mutant mice compared with controls. (Fig 7 B). (Lines368-369) We have made the following supplements in discussion: The mutant mice exhibited a significantly thickened RPE layer compared with the control group. Given the multifaceted physiological roles of RPE cells, their dysfunction and degeneration are strongly implicated in the pathogenesis of multiple retinal degenerative disorders, including retinitis pigmentosa, Best disease, age-related macular degeneration, and Stargardt disease [35]. Best’s disease (BD), an autosomal dominant macular degeneration, is characterized by the accumulation of lipofuscin-like material within and beneath the retinal pigment epithelium (RPE), driving progressive central vision loss[36].In AMD, RPE dysfunction drives the progressive accumulation of basal deposits (drusen), leading to hypoxia, choroidal neovascularization, and eventual degeneration of the RPE and photoreceptor cells[37].In this study, the mutant mice also exhibited abnormalities in the RPE. The observed RPE thickening resembles the structural changes seen in related retinal diseases, further demonstrating that the mutant mice have defects in eye development which lead to impaired visual function. (Lines451-463)

Ocular developmental abnormalities in the mutant mice lead to pupillary atresia, which prevents mydriasis. The operational principle of optical coherence tomography (OCT) depends on adequate light penetration into the fundus following mydriasis, allowing for the visualization and quantitative measurement of the retinal layered structures. The inability to induce mydriasis in the mutant mice rendered effective light entry into the fundus unattainable, making OCT measurements unfeasible in this study; consequently, data on the thickness of individual retinal layers could not be obtained. This finding aligns with the ERG results, as the abnormal pupil morphology resulted in atypical ERG waveforms. Additionally, subsequent HE pathological findings confirmed that the mutant mice exhibit structural abnormalities in the retina. We have also included microscopic images taken before and after mydriasis. As illustrated in the figure below, the pupil dilation in the C57 strain appears normal following mydriasis; however, the pupil of the model mouse shows no change, rendering OCT undetectable.

Reviewer #2:

Genetics: the breeding scheme that was used to generate the line needs to be reported here, in order to substantiate the claim that micropthalmia is heritable, and how the trait segrates. Is it consistent with a recessive allele at a single locus? is there variable penetrance and expressivity of the phenotype?

Author’s Response: We sincerely appreciate the reviewer’s critical and insightful suggestion, as clarifying the breeding scheme and genetic inheritance pattern is essential to substantiating the heritability of microphthalmia. Therefore, we have made the following supplements in the revised manuscript: A spontaneous microphthalmic mutant mouse was identified as the founder of the study. This founder was initially outcrossed with C57BL/6J mice to generate the F1 generation. All F1 mice displayed normal ocular morphology, indicating the absence of microphthalmia and suggesting that the mutant trait is not dominant. Phenotypically normal F1 heterozygous mice were intercrossed to produce the F2 generation. To confirm the inheritance patterns, F1 heterozygous mice were backcrossed with this founder, resulting in the BC1 generation. Selective intercrossing of microphthalmic mice from the F2 generation was conducted, and this inbreeding was maintained for 10 consecutive generations (F3–F10) to stabilize the phenotype until consistent microphthalmia expression was achieved in the same litter.

Statistical analysis of the F2 generation indicated that 45 out of 186 offspring displayed microphthalmia, resulting in a normal to mutant ratio of approximately 3:1 (χ² test, P > 0.05). This observation aligns with Mendelian inheritance patterns of a single autosomal recessive locus. Furthermore, the backcross (BC1) generation revealed that half of the microphthalmic mice were present, providing additional evidence that the trait is governed by a recessive allele, as homozygosity for the mutant allele is necessary for phenotypic expression.

We systematically evaluated penetrance and expressivity across the F2–F10 generations. All homozygous mutant mice displayed the microphthalmia phenotype, and no phenotypically normal mice were identified among the genetically homozygous mutants. This finding indicates complete penetrance (100%) of the trait. To assess phenotypic consistency, we quantified key ocular parameters, including equatorial diameter and lens diameter, in 30 homozygous mutant mice from the F5–F10 generations. No significant variation in the severity of microphthalmia was detected within the same litter or across generations. This observation demonstrates consistent expressivity of the phenotype, with no variability in severity. (Lines151-176)

Contribution to the field: there is a large body of literature on the genetics of retinal development, especially in the mouse model, but very little attempt here (with only two mutant mouse models mentioned) to relate the phenotype described here to the phenotypes of published mouse mutants. For example, the retinal rosettes are referred to as “typical” in discussion. What does this mean? Have they been reported previously, and how frequently? What are the genes that are linked to rosettes, and what might this suggest about the developmental processes that are disrupted?

Author’s Response: We sincerely appreciate your insightful and constructive comments, which have helped us significantly strengthen the relevance and depth of our study’s discussion. We fully agree with your observation that linking our mutant phenotype to previously reported mouse models and clarifying the biological context of retinal rosettes is critical to highlighting the study’s contribution to the field of retinal development genetics.

To address these points comprehensively, we have revised and expanded the Discussion section to systematically connect our findings with existing literature, clarify the definition and significance of retinal rosettes, and elaborate on their associated genes and underlying developmental disruptions: First, regarding the association between our mutant phenotype and published mouse models: As you noted, retinal development-related genetic studies (especially in mouse models) are extensive, and we have now explicitly bridged our observed retinal rosettes and structural abnormalities to well-characterized mutants. Specifically, we supplemented comparisons with the *Mthfr*⁺/⁻*rd8*/*rd8* mouse model [28], which exhibits focal retinal dysplasia with exaggerated retinal rosettes—an phenotype highly analogous to that in our study. Notably, the *rd8* mutation (linked to *Crb1* gene variants) is associated with Leber congenital amaurosis (LCA) and retinitis pigmentosa (RP), and *MTHFR* deficiency exacerbates this retinal disorganization [28]. Our mutant’s retinal rosettes and dysplastic features share key pathological similarities with these models, suggesting potential involvement of conserved pathways regulating retinal lamination and cell polarity. Additionally, we referenced Iwabe et al.’s work [27] on oculo-skeletal dysplasia and acquired/inherited retinal dysplasia in dogs, where retinal rosettes (characterized by outer nuclear layer invaginations) are a defining hallmark—further supporting that our observed phenotype aligns with a well-documented pattern of retinal developmental impairment across species.

Second, to clarify the term “typical” when describing retinal rosettes: We have revised this section to provide explicit context: **Definition**: Retinal rosettes are pathognomonic (characteristic) structural abnormalities of retinal dysplasia, morphologically manifesting as circular or oval invaginations/folds within the outer nuclear layer, with photoreceptor cells radially arranged around a central lumen [27,28].**Previous reports**: Such rosettes have been extensively documented in various mouse models of inherited retinal diseases, including LCA, RP, and congenital retinal dysplasia [28–30]. For instance, mutations in genes such as *Crb1* (linked to *rd8*), Nrl and Mthfr—key regulators of retinal cell proliferation and differentiation—consistently induce rosette formation [28-30]. Their occurrence is not universal but is a frequent phenotypic outcome of disrupted retinal developmental programs, particularly those governing cell polarity and laminar organization. **Implication for developmental processes**: Retinal rosette formation directly reflects perturbations in core developmental pathways: (1) impaired cell polarity (e.g., disruption of the *Crb1*-mediated apical-basal polarity complex), which is essential for maintaining retinal layer integrity; (2) dysregulated cell proliferation and differentiation, leading to disorganized retinal neuroepithelial cell arrangement; and (3) defective photoreceptor maturation or migration, resulting in the characteristic radial clustering of outer nuclear layer cells. These pathways are conserved in mammalian retinal development, and our mutant’s rosette phenotype thus suggests that the underlying causal factor (to be identified via subsequent genetic mapping) likely impacts one or more of these critical processes.

All these additions have been integrated into the revised discussion to better contextualize our findings within the broader field of retinal development genetics: Such rosettes have been extensively documented in various mouse models of inherited retinal diseases. Retinal folds exhibit structural similarities to those found in syndromic oculo-skeletal dysplasia and present as invaginations and rosettes within the outer nuclear layer, which can be detected using spectral-domain optical coherence tomography (sdOCT) as well as in conventional H&E-stained sections from dogs affected by either acquired or inherited forms of the condition [27]. In the rd8 mouse model, CRB1—expressed in Müller glia and localized to the subapical region near the outer limiting membrane—is mutated, leading to disrupted retinal lamination and the formation of folds or pseudorosettes between the outer and inner nuclear layers [28]. MTHFR deficiency exacerbates the retinal pathology in Mthfr+/−rd8/rd8 mice—a model associated with Leber congenital amaurosis and retinitis pigmentosa—manifesting as focal dysplasia featuring profound disorganization with exaggerated rosettes amidst otherwise preserved retinal architecture [29]. The retinal rosettes and dysplastic features observed in our mutant exhibit significant pathological similarities to those in these models, indicating a possible involvement of conserved pathways that regulate retinal lamination and cell polarity. In the Nrl knockout mouse, loss of neural retina leucine zipper (Nrl) function disrupts photoreceptor fate determination, causing rod precursors to undergo ectopic differentiation into cone-like cells and leading to the characteristic formation of retinal rosettes [30]. Mutations in other genes such as Dll1, Abca4, Nr2e3and PALS1 result in the formation of retinal rosettes [31-34], which is a phenotypic outcome of disrupted retinal developmental programming. The presence of retinal rosettes may indicate disturbances in fundamental developmental pathways. (Lines427-450)

“Cheryl Y. et al. demonstrated that the localized administration effectively ameliorated ocular abnormalities,including corneal, lens, and retinal defects[24]. “ - administration of what?

Author’s Response: Thank you for identifying this critical oversight. We apologize for the incomplete description and have revised the sentence to clarify the administered agent, along with key experimental details, to enhance accuracy. we have made the following supplements in the revised manuscript: Cheryl Y. et al. demonstrated that the localized administration，START (0.9% sodium chloride, 1% Tween 80, 1% powdered ataluren, 1% carboxymethylcellulose), effectively ameliorated ocular abnormalities, including corneal, lens, and retinal defects in small eye (Pax6Sey+/–) model of aniridia [24]. (Lines402-405)

“ Compared to gene knockout, spontaneous mutations more closely resemble natural pathological processes.” Please define “natural pathological processes” as this isn’t a common term.

Author’s Response: Thank you for your insightful comment. We acknowledge that the term “natural pathological processes” is not commonly used in the field and may lack clarity. We have revised the sentence to eliminate ambiguity, while retaining the core scientific premise, and supplemented contextual details to enhance understanding. We have made corrections in the revision of the manuscript：Spontaneous mutations, as naturally occurring events, produce a full and unbiased array of mutation types – single nucleotide variants (SNVs), small insertions or deletions [40]. Consequently, spontaneous mutations can lead to a variety of effects on protein function, more accurately reflecting the mutations associated with human genetic diseases than the genetically engineered knockout mutations targeting the same gene [40]. (Lines472-477)

The statement “This mutant strain represents a valuable resource for investigating spontaneous genetic defects in ocular development” is premature, as it is not supported by the evidence.

Author’s Response: Thank you for your suggestion. We realize that our expression was incorrect. We have restated it. The supplementary content in the revised manuscript is as follows: The spontaneous microphthalmia mutant model established in this study provides a reliable and clinically relevant platform for investigating the pathological mechanisms of congenital ocular developmental defects. This model recapitulates key features of human microphthalmia (e.g., reduced ocular biometrics, retinal dysplasia, and visual function deficits) and avoids the potential biases of genetically engineered models, offering a unique tool to explore naturally occurring genetic perturbations in ocular development. (Lines507-515)

Have the authors made all data underlying the findings in their manuscript fully available?

Author’s Response: We sincerely appreciate your inquiry regarding data availability. All raw data underlying the findings reported in this manuscript have been made fully available as supporting information.

Once again, I sincerely thank the editor and review experts for their hard work and valuable suggestions. We believe these supplements will make the study more comprehensive and rigorous. Thank you again for your constructive suggestions, which have helped us improve the quality of the manuscript. We hope that the revised manuscript can gain your approval. We look forward to hearing your further feedback.

Kind regards,

Jianying Wang
